# Differences in Long COVID severity by duration of illness, symptom evolution, and vaccination: a longitudinal cohort study from the INSPIRE group

**DOI:** 10.1016/j.lana.2025.101026

**Published:** 2025-02-14

**Authors:** Michael Gottlieb, Huihui Yu, Ji Chen, Erica S. Spatz, Nicole L. Gentile, Rachel E. Geyer, Michelle Santangelo, Caitlin Malicki, Kristyn Gatling, Sharon Saydah, Kelli N. O'Laughlin, Kari A. Stephens, Joann G. Elmore, Lauren E. Wisk, Michelle L'Hommedieu, Robert M. Rodriguez, Juan Carlos C. Montoy, Ralph C. Wang, Kristin L. Rising, Efrat Kean, Jonathan W. Dyal, Mandy J. Hill, Arjun K. Venkatesh, Robert A. Weinstein

**Affiliations:** a Department of Emergency Medicine, Rush University Medical Center, Chicago, IL, USA; b Section of Cardiovascular Medicine, Yale School of Medicine New Haven, CT, USA; c Yale Center for Outcomes Research and Evaluation, New Haven, CT, USA; d Department of Family Medicine, University of Washington, Seattle, WA, USA; e Department of Laboratory Medicine and Pathology, University of Washington, Seattle, WA, USA; f Department of Emergency Medicine, Yale School of Medicine, New Haven, CT, USA; g Division of Infectious Diseases, Department of Medicine, Rush University Medical Center, Chicago, IL, USA; h National Center for Immunizations and Respiratory Diseases, Centers for Disease Control & Prevention, Atlanta, GA, USA; i Departments of Emergency Medicine and Global Health, University of Washington, Seattle, WA, USA; j Division of General Internal Medicine and Health Services Research, David Geffen School of Medicine at UCLA, USA; k Department of Emergency Medicine, University of California, San Francisco, CA, USA; l Department of Emergency Medicine, Sidney Kimmel Medical College, Philadelphia, PA, USA; m Center for Connected Care, Thomas Jefferson University, Philadelphia, PA, USA; n Department of Emergency Medicine, Johns Hopkins University School of Medicine, USA; o Department of Emergency Medicine, McGovern Medical School at UTHealth Houston, USA; p Department of Medicine, Cook County Hospital, Chicago, IL, USA

**Keywords:** SARS-CoV-2, Long COVID, PROMIS, Physical health, Mental health

## Abstract

**Background:**

Although short-term outcomes of Long COVID have been described, longer-term physical and mental health outcomes of Long COVID are less well-established. This study sought to assess differences in long-term physical and mental health outcomes extending up to three years among those with current, resolved, and no Long COVID, as well as duration of Long COVID and vaccination status.

**Methods:**

This was a prospective, multisite, study of participants with SARS-CoV-2 infection from 12/7/2020-8/29/2022, with data collected through 4/2/2024. Surveys included validated tools for physical and mental health. Data were analyzed by Long COVID status (never-had, resolved, current), Long COVID duration and vaccination status.

**Findings:**

Of 3663 participants, 2604 (71.1%) never had Long COVID, 994 (27.1%) reported current Long COVID, and 65 (1.8%) reported resolved Long COVID. Compared to never having Long COVID, current Long COVID had lower/worse scores for Patient-Reported Outcomes Measurement Information System (PROMIS) version 29 Physical (7.8; 95% confidence interval [CI] 7.3–8.3) and Mental Health (9.4; 95% CI 8.8–10.1) and higher likelihood of moderate-to-high stress (adjusted odds ratio [aOR]: 2.0; 95% CI 1.6–2.4), moderate-to-high loneliness (aOR: 1.6; 95% CI 1.4–2.0), moderate-to-severe fatigue (aOR: 3.0; 95% CI 2.5–3.7), insufficient activity (aOR for Speedy Nutrition and Physical Activity Assessment ≤4: 0.6; 95% CI 0.5–0.7; aOR for Exercise Vital Sign ≤150 min/week: 0.7, 95% CI 0.6–1.0), and worse dyspnea (aOR: 5.0; 95% CI 4.3–5.8). Resolved Long COVID had lower scores for PROMIS Physical by 2.0 (95% CI 0.2–3.8) and Mental Health by 2.3 (95% CI 0.2–4.4) than the never-had-Long COVID cohort. Number of COVID-19 vaccinations was associated with better outcomes across all measures.

**Interpretation:**

Among participants followed up to 3 years after initial infection, those with current Long COVID had worse physical and mental health outcomes. The majority of those with Long COVID did not resolve, with less than 2% having resolved Long COVID. The resolved Long COVID cohort had moderately worse physical and mental health compared with those never-having-Long COVID. COVID-19 vaccination was associated with better outcomes.

**Funding:**

10.13039/100000030Centers for Disease Control and Prevention.

**Research in context**

**Evidence before this study**

Research has demonstrated the occurrence of prolonged symptoms after an initial infection with SARS-CoV-2, often referred to as Long COVID. However, much of this research has primarily focused on shorter-term outcomes of Long COVID, with most studies evaluating outcomes at 3–6 months. Data have demonstrated that symptoms evolve over time and it is believed that symptoms in the shorter-term may not reflect those later in the Long COVID condition. Despite this, there remains limited research focusing on longer-term outcomes and scant research among those who have reported resolution of their Long COVID illness. Therefore, there is a need to better understand the longer-term outcomes among those with Long COVID, as well as the influence of Long COVID length and vaccination on the longer-term outcomes. We conducted a search of PubMed using terms of “COVID”, “Long COVID”, “SARS-CoV-2”, “post-infectious sequelae”, and “longer-term” in October 2024. While we identified many studies on Long COVID outcomes, most studies included data from 3 to 12 months, with limited data beyond this period.

**Added value of this study**

We followed a population affected with SARS-CoV-2 for up to 3 years after initial infection and analyzed differences in physical and mental health among those with current, resolved, and no history of Long COVID. We further analyzed this by length of Long COVID and COVID-19 vaccination. A key strength of this study was prospective data collection using validated tools obtained directly from participants. Another strength was the length of the study, allowing key findings regarding the physical and mental health status among those with Long COVID extending to 3 years. Additionally, we were able to analyze physical and mental health among those with resolved Long COVID, which is an understudied population. Finally, we analyzed differences by length of Long COVID and vaccination dose number, to identify key predictors of illness severity.

**Implications of all the available evidence**

The results of our study add to the understanding of Long COVID by providing data on the physical and mental health outcomes among those with Long COVID extending to 3 years after their initial SARS-CoV-2 infection. The overall rate of Long COVID resolution was less than 2% in our population, with a mean duration of over 2 years. We identified significantly worse physical and mental health outcomes among those with current Long COVID, while those with resolved Long COVID had worse physical and mental health outcomes than those who never had Long COVID. We also identified better long-term outcomes among those receiving COVID-19 vaccination, that was higher among those receiving more total vaccinations. Understanding these differences is crucial to developing targeted interventions and improving patient care.

## Introduction

The World Health Organization has reported over 775 million cases of COVID-19 worldwide.^1^ Among these, approximately 11% develop persistent symptoms lasting at least 4–12 weeks after their initial severe acute respiratory syndrome coronavirus-2 (SARS-CoV-2) infection, a condition that can have substantial impact on quality-of-life and return-to-work, commonly referred to as Long COVID.^2^, ^3^, ^4^, ^5^, ^6^

Symptoms of Long COVID can vary substantially by type, frequency, and severity, and can change markedly over time in the short-term.^7^, ^8^, ^9^, ^10^, ^11^, ^12^, ^13^, ^14^, ^15^ However, Long COVID estimates often do not account for factors such as duration over the longer-term and the effect of vaccination, which can limit our understanding of Long COVID and how it may vary across groups. Additionally, data on the long-term outcomes of Long COVID are limited, particularly for those who have recovered from Long COVID, and how these individuals compare to those with current Long COVID and those who have never had Long COVID. Finally, there remain limited data on the long-term outcomes of those experiencing Long COVID and the effect on their physical and mental health using validated tools.^16^ Understanding these differences is crucial to developing targeted interventions and improving patient care.

To address these gaps, we examined differences in key patient-relevant outcomes among those with prior SARS-CoV-2 infection who had current Long COVID compared with those who never had Long COVID and those with resolved Long COVID. As a secondary analysis, we analyzed the difference in health outcome by current duration of Long COVID and the potential protective effect of COVID-19 vaccination on long-term outcomes.

## Methods

### Study design

The Innovative Support for Patients with SARS-CoV-2 Infections Registry (INSPIRE) is a prospective, longitudinal study conducted across eight major healthcare institutions in the United States intentionally selected for diversity of geographic location and participant populations (Supplementary Material).^17^ The study is registered on Clinicaltrials.gov (NCT04610515). The initial study cohort included 6044 U.S. adults with COVID-like symptoms, regardless of SARS-CoV-2 test results, who enrolled in-person or virtually between 12/7/20 and 8/29/22. Participants were enrolled (virtually or in-person) if they met the following inclusion criteria: age ≥18 years, fluency in English or Spanish, self-reported symptoms suggestive of SARS-CoV-2 (e.g., fever, cough) at time of testing, and testing with an Food and Drug Administration-approved/authorized molecular or antigen-based assay within the preceding 42 days. Exclusion criteria included inability to provide consent, being lawfully imprisoned, inability of study team to confirm the result of the index diagnostic test for SARS-CoV-2, previous SARS-CoV-2 infection >42 days before enrollment, and lacking access to an internet-connected device (e.g., smartphone, tablet, computer) for electronic survey completion.^17^

Most participants were recruited from an outpatient or emergency department population. Participants completed baseline and quarterly electronic surveys and shared electronic medical records via a patient-portal. Participants completed a final, long-term survey from 2/27/24-4/2/24, which was 18–40 months after initial SARS-CoV-2 infection. This was a cross-sectional analysis of the Long COVID-related symptoms among those who completed this final survey. Eligible participants included those who were not withdrawn or deceased at the end of the original study and did not opt out of study extension communications. Participants had 28 days to complete the survey after completing the consent and received $100 for survey completion. Surveys were collected via REDCap and sent via email or text, based on participant preference.

This analysis included INSPIRE participants who completed the consent addendum and long-term survey. To enable comparisons of participants with and without Long COVID, we restricted our cohort to those who reported ≥1 SARS-CoV-2 infection.

This study was funded by the Centers for Disease Control and Prevention (CDC). This study was reviewed and approved by the Rush University, Washington University, University of California-Los Angeles (UCLA), University of California-San Francisco, University of Texas-Houston, University of Texas-Southwestern, Yale University, and Jefferson Medical School Institutional Review Board (45 C.F.R. part 46.101(c), 25 C.F.R part 56). All participants provided written informed consent. The study adhered to the Strengthening the Reporting of Observational Studies in Epidemiology (STROBE) guidelines.

### Study outcomes

Demographics (e.g., age, sex, race, ethnicity) were collected on the baseline survey at initial study enrollment; other data were collected in the long-term survey. Long COVID status was determined by the following question: “Following COVID-19 infections, some people may develop a condition called Long COVID. This is defined as having symptoms (such as fatigue, shortness of breath, brain fog, etc.) that last for more than 12 weeks or having symptoms that suddenly emerge without another explanation. This condition is called Long COVID. Do you think you have Long COVID?” (yes or no). Participants responding yes were provided with a list of previously entered dates of SARS-CoV-2 infections (month and year) and asked to select after which infection their Long COVID symptoms first began. Participants were asked to report Long COVID status (current, resolved, never), date of onset, trajectory (improved, unchanged, waxing-and-waning, worse), and prior SARS-CoV-2 vaccination. Long COVID duration was calculated as difference in months between Long COVID onset (primary infection after which Long COVID symptoms began) and survey completion date. We intentionally used self-report of Long COVID to be consistent with the more recent approaches, which emphasize the multitude of potential symptoms and important role of patient involvement in defining Long COVID.^18^

We assessed eight patient-reported outcome measures (PROMs) as indicators of participant physical and mental health status. For physical health, we collected: Patient-Reported Outcomes Measurement Information System (PROMIS)-29 version 2.1 Physical Health global score,^19^ Fatigue Severity Scale (FSS),^20^ Speedy Nutrition and Physical Activity Assessment (SNAP),^21^ Exercise Vital Sign (EVS),^22^ and the Modified Medical Research Council (MMRC) Dyspnea scale.^23^ For mental health status, we used the PROMIS-29 version 2.1 Mental Health global score,^19^ Perceived Stress Scale (PSS),^24^ and the revised UCLA Loneliness Scale.^25^

PROMIS-29 uses a T-score metric, where a 50 represents the mean score of a reference population with a standard deviation of 10.^26^^,^^27^ For PROMIS-29 Physical Health and Mental Health scoring, higher scores correspond to lesser severity (i.e., higher scores are better). Based upon existing literature, we considered a clinically-important difference in PROMIS-29 scores to be ≥2 T-score points.^28^

FSS is a 9-item tool, with each item having a score of 1–7 (total score: 9–63), where none/mild is ≤35, moderate is 36–52, and severe is ≥53.^29^ A clinically significant FSS score is defined as ≥36.^29^

SNAP is a single-item tool asking whether participants are active for ≥30 min on five days of the week, with examples of activity including walking, housework, yardwork, dancing, or other forms of exercise. Responses range from 1 (“no, and I have no plans to be more active”) to 4 (“yes, I am active for 30 min on 5 days of the week”). We considered a SNAP score of 4 to be sufficient activity, and any score <4 to be reduced activity.

EVS is a two-item tool which quantifies number of minutes per week of moderate-to-strenuous exercise (e.g., brisk walk) using the product of two questions: number of days exercise per week (range: 0–7 days) and number of minutes exercise per day. For EVS, we used the CDC recommendation of 150 min/week, with ≥150 min/week considered sufficient and any number <150 min/week considered reduced exercise ability.^30^ For both SNAP and EVS, higher scores suggest greater degrees of physical activity.

MMRC Dyspnea scale is a single-item tool which asks participants to select when they become short of breath, ranging from 0 (“I only get breathless with strenuous exercise”) to 4 (“I am too breathless to leave the house” or “I am breathless when dressing”). Higher MMRC Dyspnea scores suggesting more severe impairment from dyspnea.

PSS is a 10-item tool, with each item ranging from 0 (never) to 4 (very often). A score of 0–13 is considered low stress, 14–26 is moderate stress, and 27–40 is high stress.

UCLA Loneliness Scale is a 20-item tool, with each item ranging from 1 (never) to 4 (often). A score of 20–34 is considered low loneliness, 35–49 is moderate loneliness, 50–64 is moderately-high loneliness, and 65–80 is high loneliness.

### Statistical analysis

For the primary analysis, we examined the difference in participants' demographics and PROM scores by Long COVID status. We conducted Kruskal–Wallis test for continuous variables and chi-square test or Fisher's exact test for categorical variables. To evaluate the impact of resolved and current Long COVID on participants' physical and mental health status, we selected different statistical models based on the distribution of the PROMs. For PROMIS-29 physical and mental health T-scores, we employed linear regression models. To ensure robustness of the model, we applied bootstrapping with 1000 replications to obtain the 95% confidence intervals (CIs). The bootstrapped intervals closely aligned with those derived directly from the linear model (Supplementary Table S1). FSS, EVS, PSS, and the UCLA Loneliness Scale had continuous scores with thresholds for levels of clinical severity. We created a logistic regression model with logit link function to estimate the odds of moderate-to-high stress (PSS ≥ 14), moderate-to-high loneliness (UCLA Loneliness Scale ≥ 35), moderate-to-severe fatigue (FSS ≥ 36), and having sufficient physical activity (EVS ≥ 150 min/week). We ran linear regression models to estimate the effects of resolved and current Long COVID on these four PROMs' original continuous scores. For SNAP scores, we first ran a logistic regression model to assess the odds of having sufficient activity with a SNAP score of 4 and then ran a proportional-odds cumulative logit model to assess the original SNAP scores as an ordinal variable. For the MMRC Dyspnea scale scores, we treated this as an ordinal variable and employed the cumulative logit model.

For each of the outcome models, we ran the unadjusted version with the Long COVID status variables (resolved Long COVID, current Long COVID, never had Long COVID) and an adjusted version including age and sex. We focused specifically on accounting for age and sex, as these are two well-described factors that carry biological plausibility.

As a secondary analysis, we analyzed Long COVID duration and SARS-CoV-2 vaccination status before Long COVID onset to understand the impact of these two factors on the PROMs. For Long COVID duration, we tested finite mixture models with 1–10 components. The finite mixture model with 2 components was selected as ‘best’ based on all model fit indices, including AIC, adjusted AIC, and BIC statistics. We used the 2.5th percentile of the second component (13-month) to separate participants with Long COVID (current or resolved) by their Long COVID duration into two groups named as short Long COVID (≤12 months) and prolonged Long COVID (>12 months) (Supplementary Figure S1).

To understand overall impact of SARS-CoV-2 vaccination on PROMs, we categorized total number of self-reported vaccination doses into four groups (0, 1–2, 3–5, ≥6 doses) and plotted PROMs by vaccination dose groups.

We used SAS version 9.4 (SAS Institute Inc., Cary, NC) and R (version 4.3.3) for statistical analyses, and Excel for visualization. Given the exploratory nature of this study, no multiplicity adjustments were performed. All tests were 2-sided with a significance threshold of p-value <0.05.

### Role of the funding source

One author (SS) from the primary funder (CDC) assisted with study design and preparation of this manuscript.

## Results

Among 4119 INSPIRE participants who consented to participate in the long-term survey, 4009 completed the survey, of whom 3663 (91%) reported prior SARS-CoV-2 infection and qualified for analysis (Supplementary Figure S2). Mean age was 40 years (SD: 14) and 66.3% were female. Overall, 13.9% were Hispanic/Latino, 66.6% were White, 7.7% Black/African American, 13.6% Asian, and 9.1% self-identified as another or multiple races. Full demographics by Long COVID status are included in Table 1. Characteristics of participants who were included versus excluded is reported in Supplementary Table S2.

**Table 1:** Demographic characteristics by self-reported Long COVID status.

Demographics	Category	Total (N = 3663)	Never had LC (N = 2604)	Resolved LC (N = 65)	Current LC (N = 994)	p^a^
Age (years)	Mean (SD)	40.2 (14.2)	39.6 (14.3)	37.1 (13.8)	41.9 (13.9)	<0.0001
	18–34	1532 (41.8)	1156 (44.4)	33 (50.8)	343 (34.5)	<0.0001
	35–49	1171 (32.0)	791 (30.4)	19 (29.2)	361 (36.3)	
	50–64	659 (18.0)	440 (16.9)	9 (13.8)	210 (21.1)	
	65+	274 (7.5)	193 (7.4)	3 (4.6)	78 (7.8)	
	Missing	27 (0.7)	24 (0.9)	1 (1.5)	2 (0.2)	
Sex	Female	2429 (66.3)	1663 (63.9)	43 (66.2)	723 (72.7)	<0.0001
	Male	1067 (29.1)	828 (31.8)	17 (26.2)	222 (22.3)	
	Transgender/Non-binary/Other	62 (1.7)	33 (1.3)	3 (4.6)	26 (2.6)	
	Missing	105 (2.9)	80 (3.1)	2 (3.1)	23 (2.3)	
Ethnicity	Non-Hispanic/Non-Latino	3082 (84.1)	2235 (85.8)	50 (76.9)	797 (80.2)	<0.0001
	Hispanic/Latino	510 (13.9)	316 (12.1)	14 (21.5)	180 (18.1)	
	Missing	71 (1.9)	53 (2.0)	1 (1.5)	17 (1.7)	
Race	White	2438 (66.6)	1758 (67.5)	35 (53.8)	645 (64.9)	<0.0001
	Black or African American	281 (7.7)	173 (6.6)	9 (13.8)	99 (10.0)	
	Asian	499 (13.6)	388 (14.9)	12 (18.5)	99 (10.0)	
	Other/Multiple	335 (9.1)	214 (8.2)	4 (6.2)	117 (11.8)	
	Missing	110 (3.0)	71 (2.7)	5 (7.7)	34 (3.4)	
Total number of SARS-CoV-2 vaccine doses	Median (Q1–Q3)	4 (3–5)	4 (3–5)	4 (3–5)	3 (2–5)	<0.0001

SD, standard deviation; LC, Long COVID.

a The Kruskal–Wallis test for age and the total number of SARS-CoV-2 vaccine does, and chi-square tests or Fisher's exact test for categorical variables were conducted to obtain the p-values.

Based on self-report, 2604 (71.1%) never had Long COVID, 994 (27.1%) had current Long COVID, and 65 (1.8%) had resolved Long COVID. Among participants with current Long COVID, 47.0% reported symptoms that waxed and waned, 26.0% reported improved symptoms, 18.8% reported no change, and 8.2% reported worsened symptoms. Among participants with current or resolved Long COVID, mean Long COVID duration was 24.2 (SD 9.4) months and 25.2 (SD 8.2) months, respectively. Among participants with current Long COVID, 15.4% were vaccinated prior to Long COVID onset. Nearly all (95.1%) participants received ≥1 SARS-CoV-2 vaccine, with the majority (71.2%) reporting 3–5 doses of the vaccine.

### Association of Long COVID status with PROMs

**Fig. 1:**
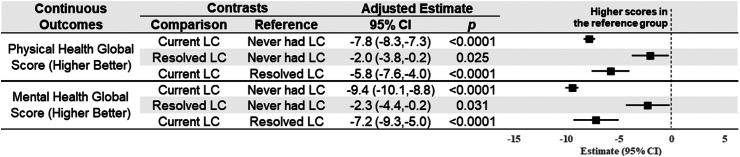
**Linear regression modeling results of the continuous outcomes for adjusted estimated differences in outcomes by Long COVID status**. Adjustment included age and sex.

**Fig. 2:**
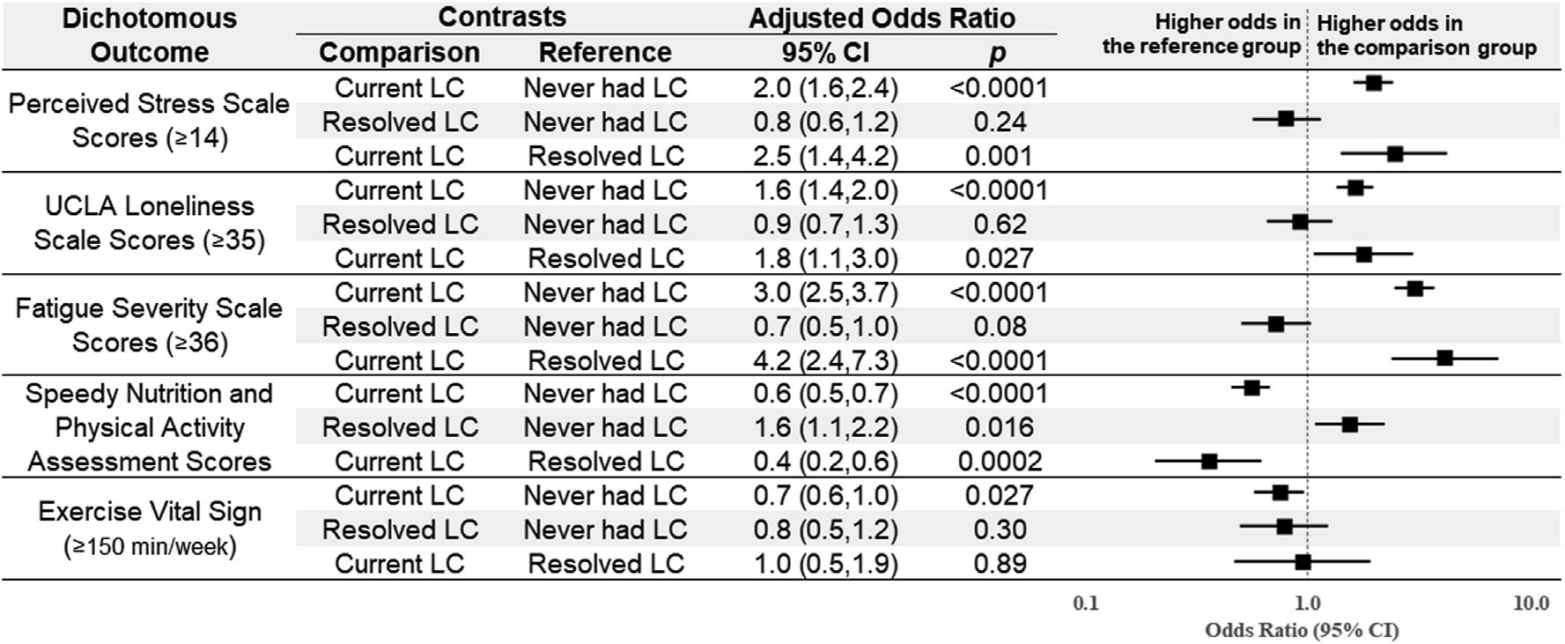
**Logistic regression modeling results of dichotomous outcomes for adjusted estimated differences in outcomes by Long COVID status**. Higher odds for perceived stress scale scores ≥14, UCLA loneliness scale scores ≥35, or fatigue severity scale scores ≥36 indicate worse outcomes; higher odds for SNAP scores = 4 or EVS ≥150 min/week indicates better outcomes; Adjustment included age and sex.

**Fig. 3:**
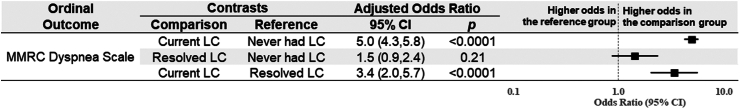
**Cumulative logit modeling results of the ordinal outcome for adjusted estimated differences in outcomes by Long COVID status**. Higher odds for higher MMRC dyspnea scale scores indicate worse outcomes; MMRC, Modified Medical Research Council; Adjustment included age and sex.

The observed (Supplementary Table S3) and modeling results (Fig. 1, Supplementary Figure S3) indicated that participants who never had Long COVID reported significantly better health status across all PROMs compared to participants with current or resolved Long COVID. The adjusted modeling results showed that the cohort without Long COVID had higher (better) PROMIS physical health scores (+7.8; 95% CI 7.3–8.3) and PROMIS mental health scores (+9.4; 95% CI 8.8–10.1) than the cohort with current Long COVID (Fig. 1). Compared to those without Long COVID, the current Long COVID cohort also had higher odds of moderate-to-high stress (adjusted odds ratio [aOR]: 2.0; 95% CI 1.6–2.4), moderate-to-high loneliness (aOR: 1.6; 95% CI 1.4–2.0), moderate-to-severe fatigue (aOR: 3.0; 95% CI 2.5–3.7), worse nutrition and physical activity scores (aOR: 0.6; 95% CI 0.5–0.7), worse EVS (aOR: 0.7; 95% CI 0.6–1.0), and worse dyspnea (aOR: 5.0; 95% CI 4.3–5.8) (Figs. 2 and 3).

When comparing those without Long COVID to those with resolved Long COVID, the resolved Long COVID cohort had lower (worse) scores on the PROMIS for physical health (−2.0; 95% CI −0.2 to −3.8) and mental health (−2.3; 95% CI −0.2 to −4.4). There was no significant difference for the other outcomes except for slightly higher odds of having better nutrition and physical activity scores in the resolved Long COVID cohort (aOR: 1.6; 95% CI 1.1–2.2). The modeling results of these PROMs in their original continuous or ordinal forms were similar (Supplementary Figure S4).

### Difference in PROMs by Long COVID duration and vaccination status

**Fig. 4:**
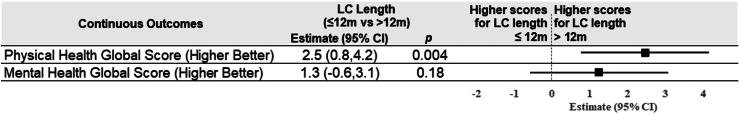
**Linear regression modeling results of the continuous outcomes for the impact of Long COVID duration among participants with current Long COVID**. Adjustment included age and sex.

**Fig. 5:**
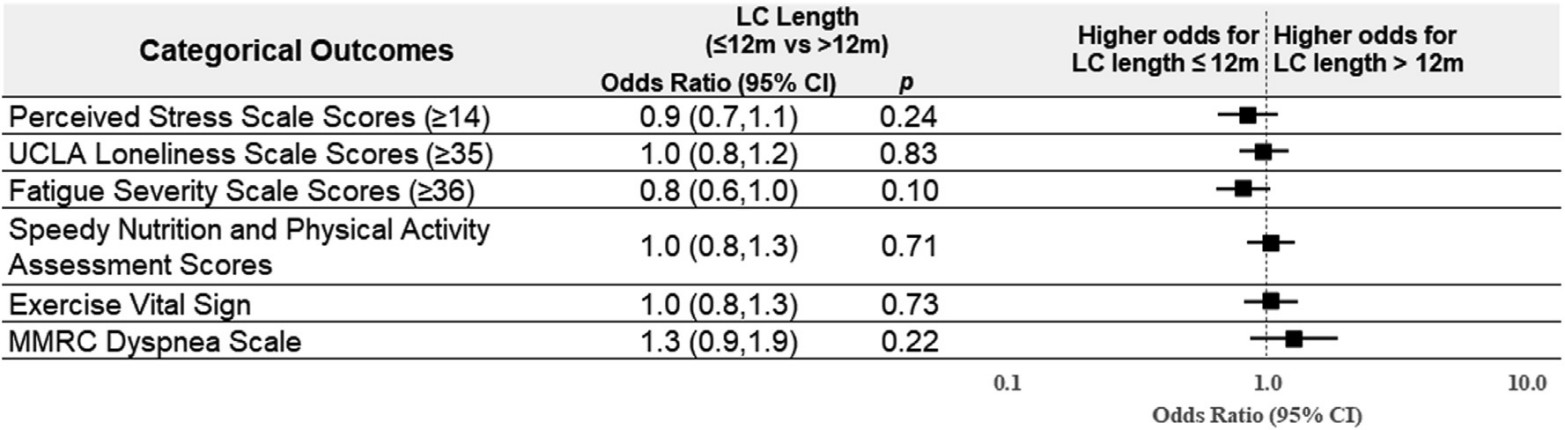
**Logistic regression modeling results of dichotomous outcomes and cumulative logit modeling results for MMRC ordinal outcome for the impact of Long COVID duration among participants with current Long COVID**. Higher odds for perceived stress scale scores ≥14, UCLA loneliness scale scores ≥35, or fatigue severity scale scores ≥36 indicate worse outcomes; higher odds for SNAP scores = 4 or EVS ≥150 min/week indicates better outcomes; Higher odds for higher MMRC dyspnea scale scores indicate worse outcomes; MMRC, Modified Medical Research Council; Adjustment included age and sex.

**Fig. 6:**
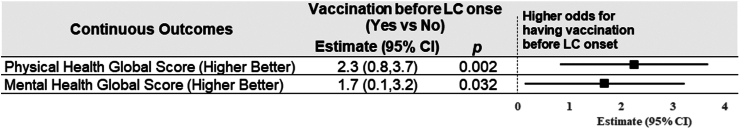
**Linear regression modeling results of the continuous outcomes for the impact of COVID-19 vaccination before Long COVID onset on outcomes among participants with current Long COVID**. Adjustment included age and sex.

**Fig. 7:**
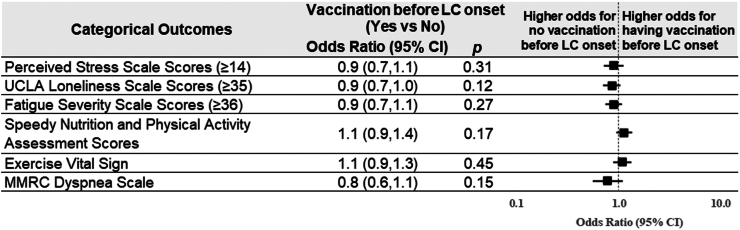
**Logistic regression modeling results of dichotomous outcomes and Cumulative logit modeling results for MMRC ordinal outcome for the impact of COVID-19 vaccination before Long COVID onset on outcomes among participants with current Long COVID**. Higher odds for perceived stress scale scores ≥14, UCLA loneliness scale scores ≥35, or fatigue severity scale scores ≥36 indicate worse outcomes; higher odds for SNAP scores = 4 or EVS ≥150 min/week indicates better outcomes; Higher odds for higher MMRC dyspnea scale scores indicate worse outcomes; MMRC, Modified Medical Research Council; Adjustment included age and sex.

When analyzing association of Long COVID duration (≤12 months vs. >12 months) in the current Long COVID group, there was no significant difference across most outcomes except for a slightly higher (better) PROMIS physical health score in those with prolonged Long COVID (>12 months) (Figs. 4 and 5). When analyzing the association of vaccination prior to Long COVID onset with PROMs in the current Long COVID group, those who were vaccinated prior to Long COVID had higher (better) PROMIS physical health scores (+2.3; 95% CI 0.8–3.7) and PROMIS mental health scores (+1.7; 95% CI 0.1–3.2) (Figs. 6 and 7).

**Fig. 8:**
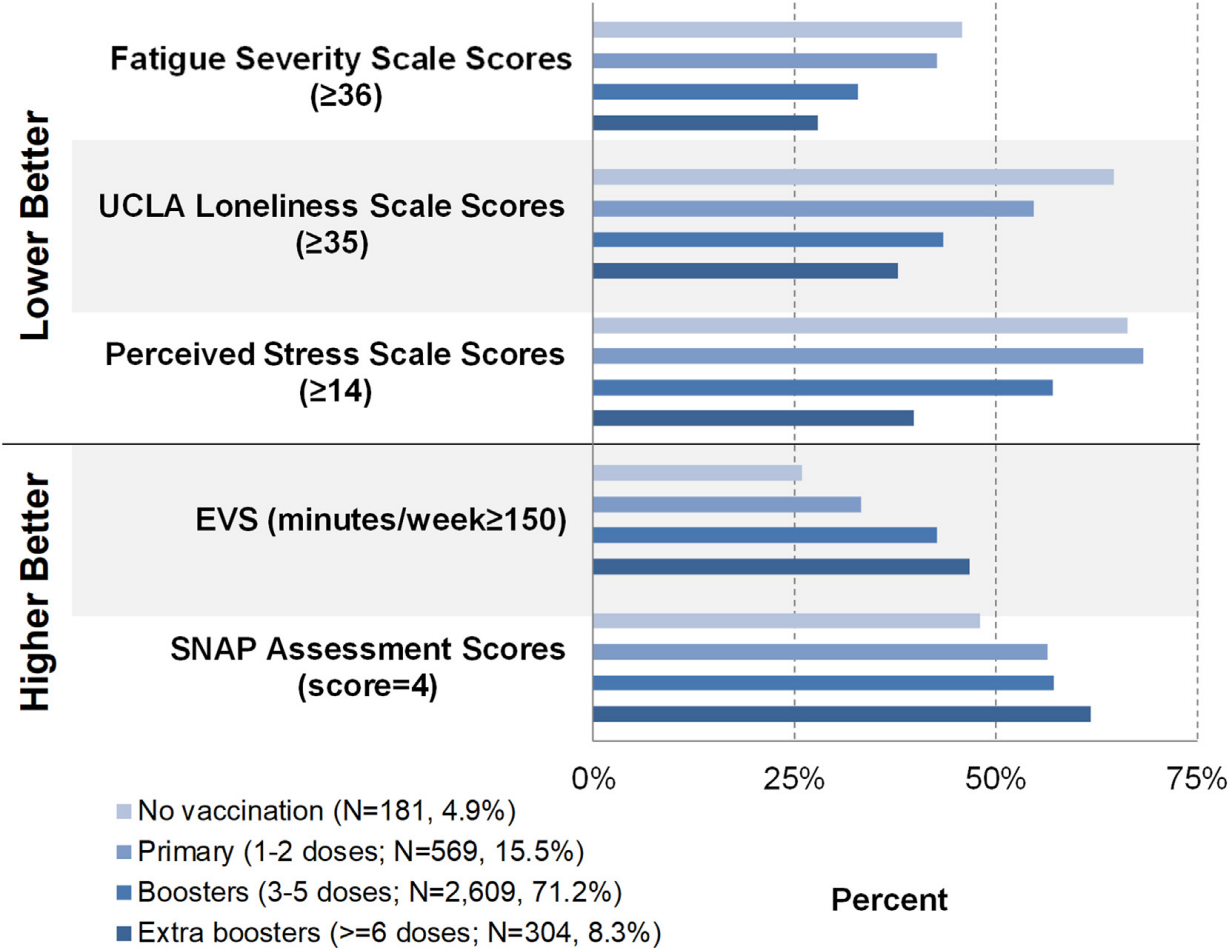
**Health outcomes among all participants by COVID-19 vaccine dose count**. EVS, exercise vital sign; SNAP, Speedy Nutrition and Physical Activity.

We plotted PROMs by vaccination dose counts across all participants and observed better PROMs, including PROMIS physical and mental health, FSS, SNAP, EVS, PSS, and UCLA Loneliness scores, for those who received more vaccine doses (0, 1–2, 3–5, ≥6 doses) (Fig. 8, Supplementary Figure S5).

## Discussion

In this prospective study of participants with SARS-CoV-2 infection, we examined long-term physical and mental health outcomes up to 40 months post-infection by participants' self-reported Long COVID status. Overall, less than 2% of participants had resolution of Long COVID, with a mean Long COVID duration of over 2 years. This highlights an important finding, suggesting that the majority of people with Long COVID may not experience complete resolution, and for those that do the time period to resolution will be long.

Additionally, participants with current Long COVID had markedly worse physical and mental health outcomes compared with those who reported never having Long COVID and those with resolved Long COVID. This is consistent with prior research demonstrating worse outcomes at 2–3 years among those with Long COVID.^16^ Importantly, while those with resolved Long COVID had better outcomes compared with those with current Long COVID, their physical and mental health outcomes remained worse compared to those who never had Long COVID. The better PROMs among those with resolved Long COVID is tempered by their incomplete improvement that warrants longer-term assessment. These findings demonstrate the substantial negative long-term impact of Long COVID, including those with current Long COVID and resolved Long COVID.

We identified key and dramatic Long COVID-associated differences across multiple physical health outcomes, including overall physical health, fatigue severity, physical activity, and dyspnea. PROMIS is a commonly used, validated tool for assessing overall physical health outcomes, with prior literature suggesting worse outcomes in those with Long COVID.^31^^,^^32^ Our study builds upon this by demonstrating continued impact on physical health outcomes in those with Long COVID extending to over three years post-infection. Fatigue is another well-described symptom which can substantially affect quality-of-life, and we found a significantly higher number of participants (66.5%) reporting severe fatigue in the current Long COVID group, as well as a marked increase compared with shorter-term assessments.^13^

Mental health outcomes were also worse in the participants reporting current Long COVID. The current Long COVID cohort also experienced greater degrees of stress and loneliness compared with those who never had Long COVID. This latter aspect may reflect the physical and mental health impacts of Long COVID leading to those with Long COVID experiencing greater degrees of stress and social isolation. As social support can help improve overall outcomes,^33^ it is possible that worse physical and mental health may also further impact their courses by limiting access to these social support structures, highlighting a critical need for greater mental health and social support structures for those with Long COVID.

While participants with resolved Long COVID experienced improvements in outcomes compared to those with current Long COVID, the resolved Long COVID cohort had worse overall physical and mental health scores than those who never had Long COVID. For example, the resolved Long COVID PROMIS scores for both physical and mental health were still at least 2 points below those without Long COVID, demonstrating a clinically significant difference in overall physical and mental health between groups.^28^ Therefore, while much of the focus has been on those experiencing Long COVID, further research is needed to evaluate the residual effects among those with resolved Long COVID, who may not actually be fully back to their pre-COVID state and warrant continued attention.

We also analyzed length of Long COVID across several key PROMs. We identified a small difference in PROMIS physical health scores among those with prolonged Long COVID (>12 months) compared with short Long COVID (≤12 months), but did not identify a difference for any other outcomes. This suggests that those with longer duration of Long COVID may experience slight improvements in physical health over time, but have an otherwise similar outcome to those with a shorter course. This is important for advising patients who fall within either cohort and for resource planning.

Vaccination prior to first SARS-CoV-2 infection was associated with better physical and mental health outcomes. This builds upon existing observational research demonstrating a potential benefit to vaccination prior to SARS-CoV-2.^34^ Subsequently, we observed markedly better physical and mental health among those receiving multiple boosters compared with no vaccination or only a primary vaccination series before SARS-CoV-2 infection. This supports a key protective effect of vaccination, which is more pronounced among those receiving booster vaccinations.

### Limitations

Long COVID status was based on self-report, rather than objective testing or specific symptom criteria, and may include conditions not caused by Long COVID. However, this is consistent with the recent approach to defining Long COVID, which emphasizes the myriad symptoms and importance of patient involvement in defining Long COVID.^18^ INSPIRE eligibility required access to an internet-capable device, which may reflect a population with more technological access. Participation in the long-term survey required completion of a consent addendum, which limited retention of the overall study cohort, although 66% participation (91% among those completing the consent addendum) remains high given the length of follow-up. Recall bias related to dates of SARS-CoV-2 infections, vaccinations, and symptom onset is also a consideration, as surveys were completed up to 40 months after first SARS-CoV-2 infection. However, participants were prompted to refer to their medical records and vaccination cards at survey start. While we used multiple tools to elucidate both the physical and mental health effects of Long COVID, it is unclear the degree to which these may influence each other (e.g., physical health impairments leading to depression and isolation). Future work should further explore this area and the degree to which these aspects influence other health outcomes. We did not account for specific SARS-CoV-2 strain during the initial infection. Additionally, we did not account for the influence of subsequent SARS-CoV-2 infections after Long COVID onset. In our modeling, we focused specifically on accounting for age and sex. Other comorbidity data, including body mass index were more limited in our data set, due to variation in reporting, and differences in diagnoses over time. As this data was collected at study enrollment and not at the time of onset of their Long COVID illness, we opted not to include this in the statistical adjustments. For modeling PROMIS physical health scores, the outcome slightly violated the normality assumption due to the nature of the PROMIS score. Specifically, a portion of participants reached the ceiling value, resulting in a skewed distribution. To ensure consistency and interpretability, we chose to retain the ordinal linear model. Moreover, as a cross-sectional analysis, we were unable to account for differences in baseline status across groups, and it is possible that some groups may have had a worse baseline health status. Those who received more vaccinations may reflect a more health-conscious population. As the original study was not specifically powered for this outcome, a post-hoc analysis was conducted. With a pooled standard deviation of 9 for the PROMIS score, alpha = 0.05, and 80% power, the sample sizes for each Long COVID status group (2604 never had Long COVID, 994 current Long COVID, and 65 resolved Long COVID) allow detection of differences as follows: 3.17 for never Long COVID vs. resolved Long COVID, 3.24 for current Long COVID vs. resolved Long COVID, and 0.94 for never Long COVID vs. current Long COVID. Due to the small sample size in the resolved group, there is reduced precision in the estimates, requiring a larger difference to achieve statistical significance. Finally, while we analyzed participants based on short versus prolonged Long COVID using finite mixture models, this was assessed at the time of the survey completion and not upon Long COVID resolution. As such, some participants in the short Long COVID cohort may simply reflect the prolonged Long COVID at an earlier time point.

### Conclusion

In this prospective study following participants up to 40 months after initial SARS-CoV-2 infection, we found the majority of participants with Long COVID did not experience resolution, with only 2% having resolved Long COVID. Among individuals reporting current Long COVID, we identified significantly worse physical and mental health compared to those never-having- Long COVID or with resolved Long COVID. Among the resolved Long COVID cohort, we identified worse physical and mental health compared with those who never had Long COVID. Among those who were vaccinated prior to their COVID-19 illness, there was improved physical and mental health outcomes with a dose-response relationship demonstrating better outcomes with more vaccinations. These findings provide valuable insights for patients, clinicians, and researchers regarding the burden of Long COVID illness as well as the importance of PROMs on the development of future interventions and treatment.

## Contributors

**Michael Gottlieb** - conceptualization, funding acquisition, investigation, methodology, project administration, resources, supervision, validation, visualization, writing – original draft, and writing – review & editing.

**Huihui Yu** - conceptualization, data curation, formal analysis, investigation, methodology, software, validation, visualization, writing – original draft, and writing – review & editing.

**Ji Chen** - conceptualization, data curation, formal analysis, investigation, methodology, software, validation, visualization, writing – original draft, and writing – review & editing.

**Erica S. Spatz** - conceptualization, investigation, methodology, project administration, resources, writing – original draft, and writing – review & editing.

**Nicole L. Gentile** - conceptualization, investigation, methodology, project administration, resources, writing – original draft, and writing – review & editing.

**Rachel E. Geyer** - conceptualization, investigation, methodology, project administration, resources, writing – original draft, and writing – review & editing.

**Michelle Santangelo** - conceptualization, investigation, methodology, project administration, resources, writing – original draft, and writing – review & editing.

**Caitlin Malicki** - conceptualization, investigation, methodology, project administration, resources, writing – original draft, and writing – review & editing.

**Kristyn Gatling** - conceptualization, investigation, methodology, project administration, resources, writing – original draft, and writing – review & editing.

**Sharon Saydah** - conceptualization, investigation, methodology, project administration, resources, writing – original draft, and writing – review & editing.

**Kelli N. O'Laughlin** - conceptualization, investigation, methodology, project administration, resources, writing – original draft, and writing – review & editing.

**Kari A. Stephens** - conceptualization, investigation, methodology, project administration, resources, writing – original draft, and writing – review & editing.

**Joann G. Elmore** - conceptualization, investigation, methodology, project administration, resources, writing – original draft, and writing – review & editing.

**Lauren E. Wisk** - conceptualization, investigation, methodology, project administration, resources, writing – original draft, and writing – review & editing.

**Michelle L'Hommedieu** - conceptualization, investigation, methodology, project administration, resources, writing – original draft, and writing – review & editing.

**Robert M. Rodriguez** - conceptualization, investigation, methodology, project administration, resources, writing – original draft, and writing – review & editing.

**Juan Carlos C. Montoy** - conceptualization, investigation, methodology, project administration, resources, writing – original draft, and writing – review & editing.

**Ralph C. Wang** - conceptualization, investigation, methodology, project administration, resources, writing – original draft, and writing – review & editing.

**Kristin L. Rising** - conceptualization, investigation, methodology, project administration, resources, writing – original draft, and writing – review & editing.

**Efrat Kean** - conceptualization, investigation, methodology, project administration, resources, writing – original draft, and writing – review & editing.

**Jonathan W. Dyal** - conceptualization, investigation, methodology, project administration, resources, writing – original draft, and writing – review & editing.

**Mandy J. Hill** - conceptualization, investigation, methodology, project administration, resources, writing – original draft, and writing – review & editing.

**Arjun K. Venkatesh** - conceptualization, investigation, methodology, project administration, resources, writing – original draft, and writing – review & editing.

**Robert A. Weinstein** - conceptualization, funding acquisition, investigation, methodology, project administration, resources, supervision, validation, visualization, writing – original draft, and writing – review & editing.

## Data sharing statement

Deidentified data will be made available upon completion of the full study and publicly accessible through the CDC.

## Declaration of interests

Michael Gottlieb reports funding from Society for Academic Emergency Medicine Foundation Emerging Infectious Disease and Preparedness Grant. Nicole L. Gentile reports funding from Improving Access to Multidisciplinary Care for Patients with Long COVID (AHRQ, U18 HS29905-01), Pain Relief with Integrative Medicine (PRIMe)?: Feasibility Trial of Acupuncture for Long COVID (NCCIH, R34AT012679-01), Interaction between SARS-CoV-2 Infection and Ancestral genomic Variations in the Risk of Alzheimer's Disease and Related Disorders (ISAVRAD) (NIA, U19 AG076581-01A1), Post-Acute Sequelae of SARS-CoV-2 (PASC): Analysis of Autoantibody Abnormalities and Impact of Pain on Quality of Life and Function Royalty Research Fund – University of Washington. Rachel E. Geyer reports funding from Improving Access to Multidisciplinary Care for Patients with Long COVID (AHRQ, U18 HS29905-01), Pain Relief with Integrative Medicine (PRIMe)?: Feasibility Trial of Acupuncture for Long COVID (NCCIH, R34AT012679-01), Post-Acute Sequelae of SARS-CoV-2 (PASC): Analysis of Autoantibody Abnormalities and Impact of Pain on Quality of Life and Function Royalty Research Fund – University of Washington UW Core Center for Clinical Research (CCCR) of Musculoskeletal Conditions (NIH, P30 AR072572-08). Joann G Elmore serves as Editor-In-Chief for adult general medicine topics at UpToDate. Ralph C Wang reports funding from the SAEM Foundation Emerging Infectious Disease and Preparedness Grant. Kristin Rising reports funding from the Preventing Emerging Infections Through Vaccine Effectiveness Testing (Project PREVENT) II (CDC U01CK00048), PROmotion of COVid-19 VA(X)ccination in the Emergency Department (PROCOVAXED; NIH 1R01AI66967), and the COVID-19 Mobile Vaccination Program – Philadelphia Department of Public Health. Arjun K Venkatesh reports funding from Society for Academic Emergency Medicine Foundation Emerging Infectious Disease and Preparedness Grant. All authors received institutional funding from the Centers for Disease Control and Prevention (75D30120C08008) for the conduct of this study.
