# Supplementary material for: Differences in Long COVID severity by duration of illness, symptom evolution, and vaccination: a longitudinal cohort study from the INSPIRE group
Source: Lancet Reg Health Am. 2025 Feb 14;44:101026. doi: 10.1016/j.lana.2025.101026 (PMC11875141; doi:10.1016/j.lana.2025.101026)
Supplement: Supplementary Tables and Figures [file mmc1.docx]

**Physical and Mental Health Outcome Differences by Long COVID Status, Duration of Illness, and Vaccination: A Longitudinal Cohort Study from INSPIRE**

**SUPPLEMENTARY MATERIAL**

**Table of Contents**

1. **Supplementary Table 1. Comparison of 95% confidence intervals for the primary outcome using the linear versus bootstrapping with 1,000 replications (Page 2)**
2. **Supplementary Table 2. Characteristics of Participants who were included and excluded in the analysis (Page 3)**
3. **Supplementary Table 3. Observed Distributions of Patient-Reported Outcome Measures by Long COVID Status (Page 4)**
4. **Supplementary Figure 1. The distribution of Long COVID duration among participants with Long COVID at any point based on principal component analysis. (Page 5)**
5. **Supplementary Figure 2. Flow diagram of enrollment.** **(Page 7)**
6. **Supplementary Figure 3. Unadjusted estimated differences in outcomes by Long COVID status**. **(Page 8)**
7. **Supplementary Figure 4.** **Estimated difference in outcomes by Long COVID status. (Pages 9)**
8. **Supplementary Figure 5. Health Outcomes Among All Participants by COVID-19 Vaccine Dose Count. (Page 10)**

**Supplementary Table 1. Comparison of 95% confidence intervals (CIs) for the primary outcome using the linear versus bootstrapping with 1,000 replications.** To assess the robustness of the modeling results for the PROMIS physical health scores, we applied bootstrapping with 1,000 replications to obtain the 95% CIs. The bootstrapped intervals closely align with those derived directly from the linear model.

| **Contrasts** | **95% CI by linear model** | **95% CI from bootstrapping** |
| --- | --- | --- |
| **Current LC vs. Never had LC** | (-8.3, -7.3) | (-8.3, -7.2) |
| **Resolved LC vs. Never had LC** | (-3.8, -0.2) | (-4.0, -0.3) |
| **Current LC vs. Resolved LC** | (-7.6, -4.0) | (-7.5, -3.8) |

***LC, Long COVID; CI, confidence interval***

**Supplementary Table 2. Characteristics of Participants who were included and excluded in the analysis**

| Demographics | Category | Excluded participant  (n=2035) | Included participants  (n=4009) | p^a^ |
| --- | --- | --- | --- | --- |
|  |  |  |  |  |
| Age (years) | Mean (SD) | 40.88 (15.36) | 40.11 (14.37) | 0.05 |
|  | 18 to 34 | 834 (41.0) | 1700 (42.4) | 0.11 |
|  | 35 to 49 | 611 (30.0) | 1256 (31.3) |  |
|  | 50 to 64 | 385 (18.9) | 723 (18.0) |  |
|  | 65+ | 190 ( 9.3) | 301 ( 7.5) |  |
|  | Missing | 15 ( 0.7) | 29 ( 0.7) |  |
| Sex | Female | 1305 (64.1) | 2667 (66.5) | 0.011 |
|  | Male | 660 (32.4) | 1158 (28.9) |  |
|  | Transgender/  Non-binary/Other | 27 ( 1.3) | 66 ( 1.6) |  |
|  | Missing | 43 ( 2.1) | 118 ( 2.9) |  |
| Ethnicity | Non-Hispanic | 1657 (81.4) | 3369 (84.0) | 0.023 |
|  | Hispanic | 340 (16.7) | 563 (14.0) |  |
|  | Missing | 38 ( 1.9) | 77 ( 1.9) |  |
| Race | White | 1302 (64.0) | 2620 (65.4) | <.0001 |
|  | Black or African American | 281 (13.8) | 335 ( 8.4) |  |
|  | Asian | 203 (10.0) | 564 (14.1) |  |
|  | Other/Multiple | 178 ( 8.7) | 365 ( 9.1) |  |
|  | Missing | 71 ( 3.5) | 125 ( 3.1) |  |

*SD, standard deviation; ^a^The Kruskal Wallis test for the continuous age, and chi-square test or Fisher’s exact test for categorical variables were conducted to obtain the p-values.*

**Supplementary Table 3. Observed Distributions of Patient-Reported Outcome Measures by Long COVID Status**

| **Outcomes^a^** | **Distribution** | **Never had LC** | **Fully Resolved LC** | **Current LC** | ***p^b^*** |
| --- | --- | --- | --- | --- | --- |
| **Physical Health Global Score  (Higher is Better)** | Mean (SD) | 53.9 (6.6) | 51.9 (7.7) | 45.8 (8.5) | <.0001 |
|  | Median (Q1-Q3) | 57.2  (49.8-58.5) | 56.5  (46.0-57.6) | 45.1  (39.4-55.2) |  |
|  | % of ≥42 | 92.7 | 89.2 | 62.8 | <.0001 |
| **Mental Health Global Score  (Higher is Better)** | Mean (SD) | 53.9 (8.2) | 51.2 (8.8) | 44.2 (9.0) | <.0001 |
|  | Median (Q1-Q3) | 55.0  (48.6-60.2) | 53.8  (43.0-57.5) | 43.8  (38.2-50.6) |  |
|  | % of ≥40 | 94.2 | 86.2 | 66.1 | <.0001 |
| **Perceived Stress Scale Scores  (Lower is Better)** | Mean (SD) | 13.8 (7.3) | 15.6 (7.4) | 19.0 (7.5) | <.0001 |
|  | Median (Q1-Q3) | 14  (8-19) | 16  (9-21) | 20  (14-24) |  |
|  | % of ≥14 | 50.7 | 61.5 | 76.3 | <.0001 |
| **UCLA Loneliness Scale Scores  (Lower is Better)** | Mean (SD) | 34.1 (11.8) | 36.2 (12.1) | 41.4 (13.5) | <.0001 |
|  | Median (Q1-Q3) | 31  (25-41) | 34  (27-44) | 40  (30-51) |  |
|  | % of ≥35 | 39.2 | 49.2 | 62.9 | <.0001 |
| **Fatigue severity scale scores  (Lower is Better)** | Mean (SD) | 22.5 (16.0) | 28.6 (17.7) | 40.9 (15.8) | <.0001 |
|  | Median (Q1-Q3) | 20  (11-34) | 27  (16-40) | 43  (30-54) |  |
|  | % of ≥36 | 22.5 | 33.8 | 66.5 | <.0001 |
| **Exercise Vital Sign (10-minute/week)** | Mean (SD) | 154.8 (150.1) | 161.1 (144.8) | 116.1 (149.1) | <.0001 |
|  | Median (Q1-Q3) | 12  (6-20) | 12  (9-24) | 6  (2-15) |  |
|  | % of ≥150 | 45.2 | 44.6 | 28.9 | <.0001 |
| **Speedy Nutrition and Physical Activity Assessment Scores, n (%)** |  |  |  |  | <.0001 |
| [4] Yes, I am active for 30 minutes on 5 days of the week |  | 1,614 (62.0%) | 44 (67.7%) | 431 (43.4%) |  |
| [3] Sometimes I am active for 30 minutes, but not all the time |  | 714 (27.4%) | 13 (20.0%) | 386 (38.8%) |  |
| [2] No, but I have been thinking about being more active. |  | 257 (9.9%) | 8 (12.3%) | 167 (16.8%) |  |
| [1] No, and I have no plans to be more active. |  | 19 (0.7%) | 0 (0.0%) | 10 (1.0%) |  |
| **MMRC Dyspnea Scale, n (%)** |  |  |  |  | <.0001 |
| [1] I only get breathless with strenuous exercise |  | 1,769 (67.9%) | 38 (58.5%) | 298 (30.0%) |  |
| [2] I get short of breath when hurrying or walking up a slight hill. |  | 737 (28.3%) | 23 (35.4%) | 504 (50.7%) |  |
| [3] I walk slower than people of the same age because of breathlessness or have to stop for breath when walking at my own pace. |  | 67 (2.6%) | 2 (3.1%) | 145 (14.6%) |  |
| [4] I stop for breath after walking about 100 yards or after a few minutes. |  | 23 (0.9%) | 1 (1.5%) | 38 (3.8%) |  |
| [5] I am too breathless to leave the house" or "I am breathless when dressing". |  | 8 (0.3%) | 1 (1.5%) | 9 (0.9%) |  |

*SD, standard deviation;  ^a^Physical health global scores, mental health global scores, perceived stress scale scores, UCLA loneliness scale scores, fatigue severity scale scores, and exercise vital sign scores are continuous variables. A PROMIS physical health global score ≥ 42 indicates good-to-excellent physical global health, a PROMIS mental health global score ≥ 40 indicates good-to-excellent mental global health, a perceived stress scale score ≥ 14 indicates moderate-to-severe stress, a UCLA loneliness scale score ≥ 35 indicates moderate-to-severe loneliness, a fatigue severity scale score ≥ 36 indicates moderate-to-severe fatigue, and exercise of 150 or more minutes indicates sufficient activity. Speedy nutrition and physical activity assessment scores and MMRC Dyspnea scale scores are categorical variables.*

^b^*Kruskal Wallis tests for the continuous scores and chi-square tests or Fisher’s exact test for the probability of scores greater than a cutoff and categorical variables were conducted to obtain the p-values.*

**Supplementary Figure 1. The distribution of Long COVID duration among participants with Long COVID at any point based on principal component analysis.**


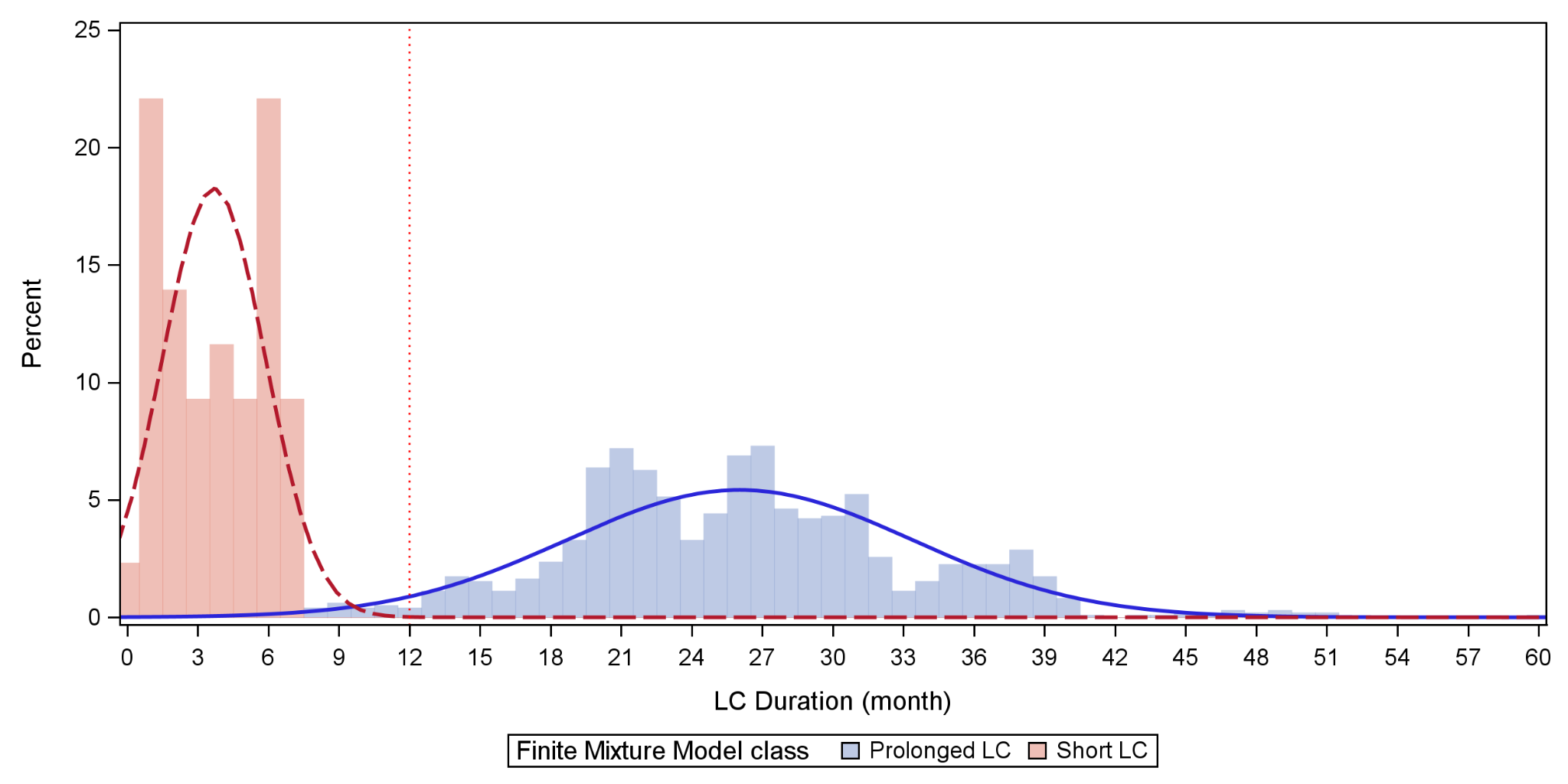


**Supplementary Figure 2. Flow diagram of enrollment.**


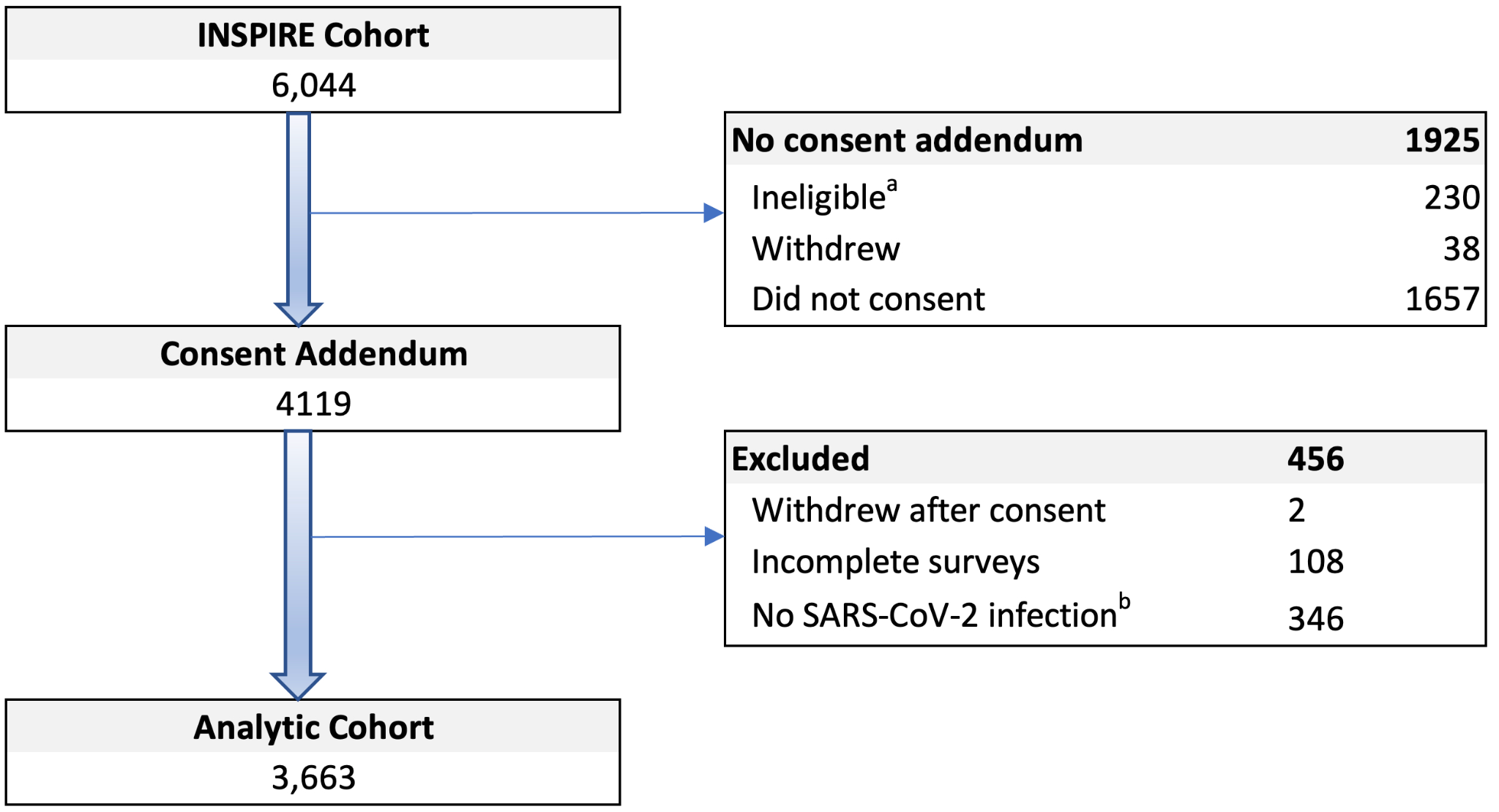


*^a^Ineligible to receive consent addendum invitation due to being withdrawn or deceased at the end of the original study phase, opted out of study extension communications or did not opt in to receive the invitation (University of Texas Southwestern site only); ^b^No SARS-CoV-2 infection reported on the long-term survey.*

**Supplementary Figure 3. Unadjusted estimated differences in outcomes by Long COVID status**

1. **Linear regression modeling results of the continuous outcomes**

**
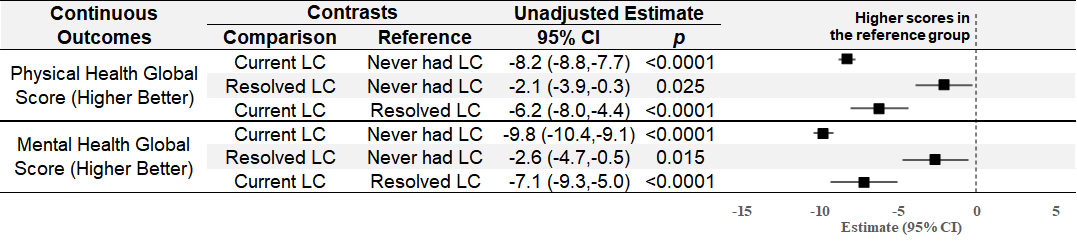
**

1. **Logistic regression modeling results of dichotomous outcomes**

**
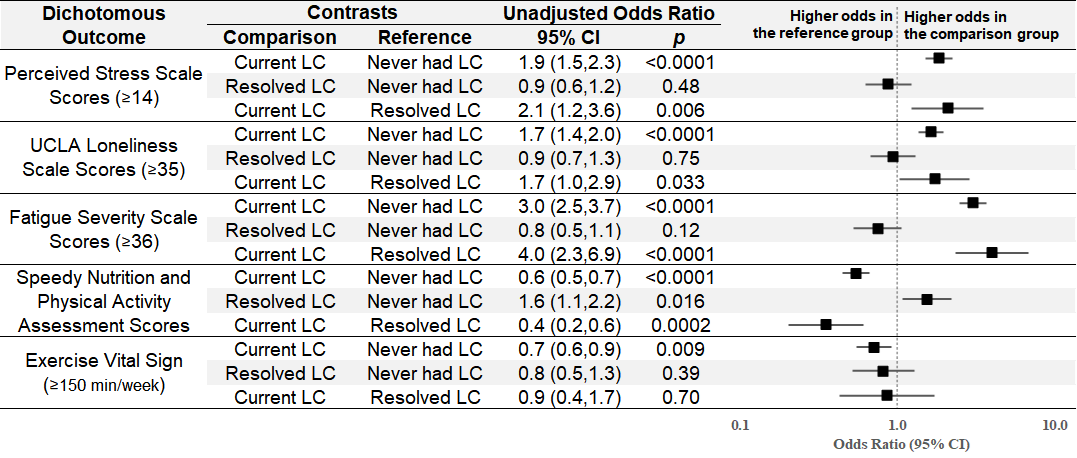
**

1. **Cumulative logit modeling results of the ordinal outcome**

**
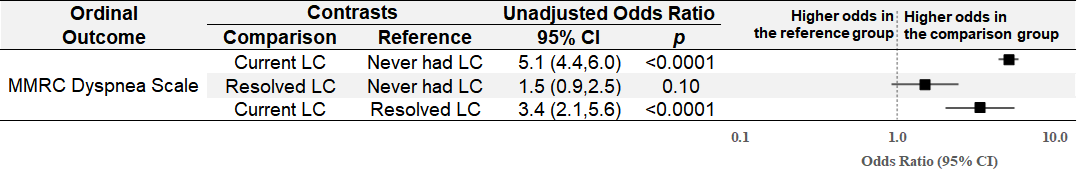
**

**Supplementary Figure 4. Estimated difference in outcomes by Long COVID status**

**a) Linear regression modeling results of the continuous outcomes**

**
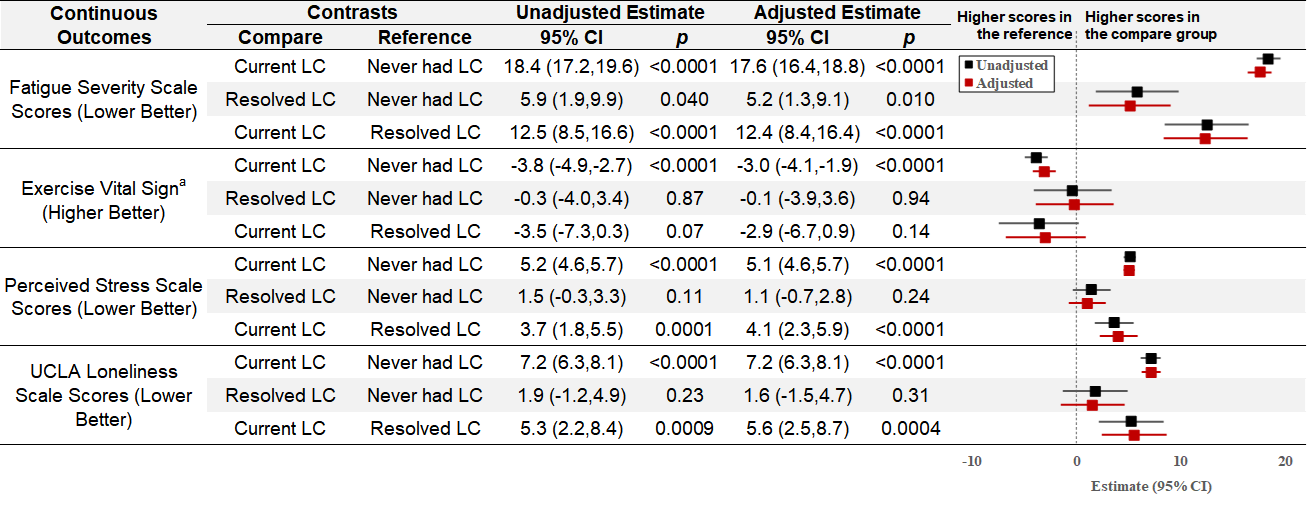
**

*^a^The unit for exercise vital sign scoring is based on 10 minutes/week. Adjustment included age and sex.*

**b) Cumulative logit modeling results of the ordinal outcome**

**
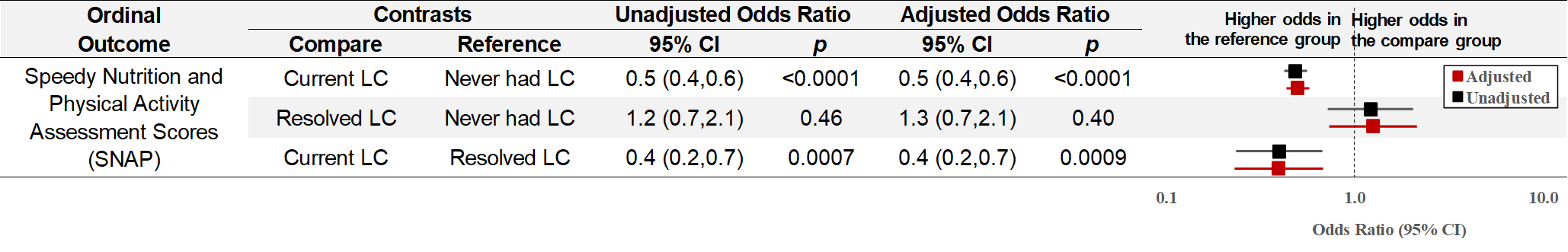
**

*Adjustment included age and sex.*

**Supplementary Figure 5. Health Outcomes Among All Participants by COVID-19 Vaccine Dose Count**


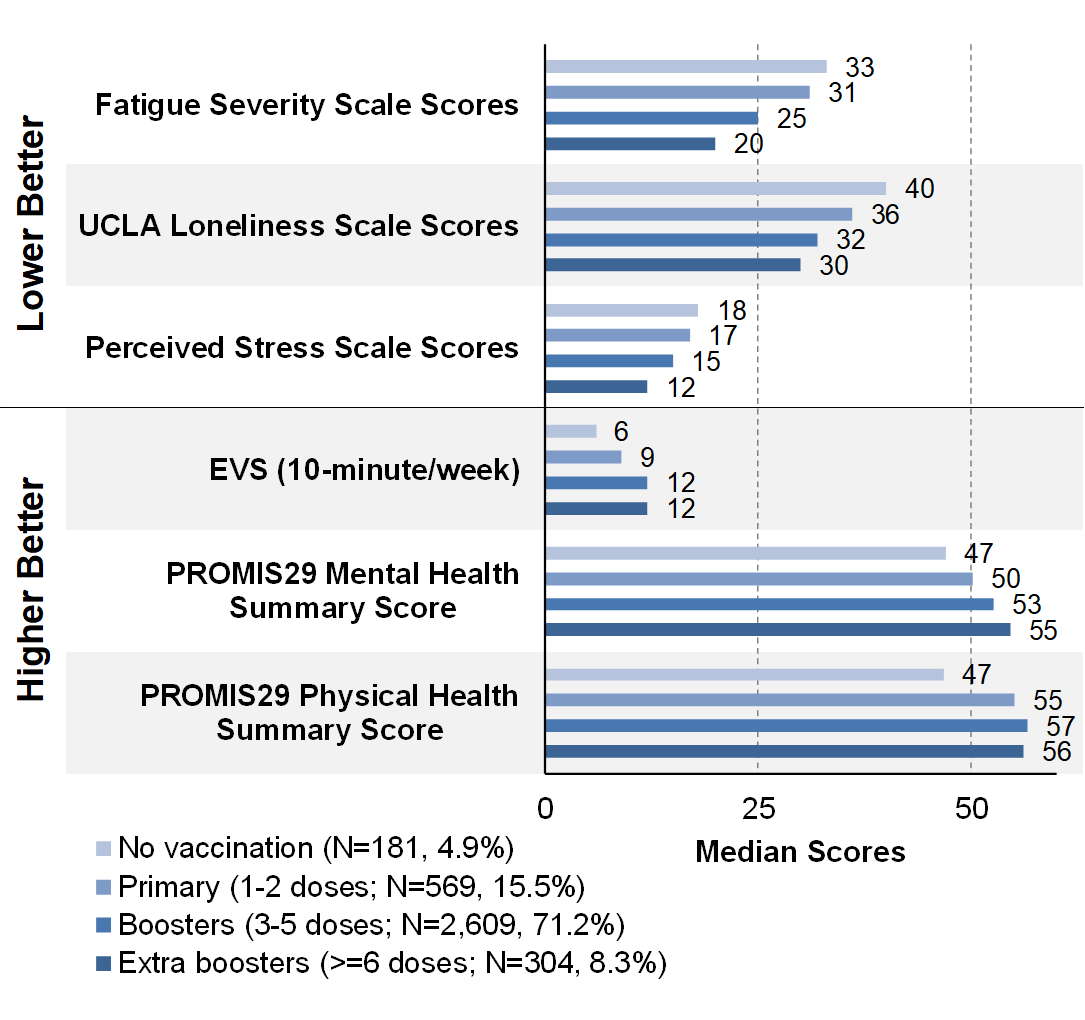


*EVS, exercise vital sign*
